# Animal blood in translational research: How to adjust animal blood viscosity to the human standard

**DOI:** 10.14814/phy2.14880

**Published:** 2021-05-27

**Authors:** Paul Ecker, Andreas Sparer, Benjamin Lukitsch, Martin Elenkov, Monika Seltenhammer, Richard Crevenna, Margit Gföhler, Michael Harasek, Ursula Windberger

**Affiliations:** ^1^ Institute of Chemical Environmental and Bioscience Engineering TU Wien Vienna Austria; ^2^ Institute of Engineering Design and Product Development TU Wien Vienna Austria; ^3^ Decentralized Biomedical Facilities Center for Biomedical Research Medical University of Vienna Vienna Austria; ^4^ Center for Forensic Medicine Medical University Vienna Vienna Austria; ^5^ University Clinic of Physical Medicine Rehabilitation and Occupational Medicine Medical University Vienna Vienna Austria

## Abstract

Animal blood is used in mock circulations or in forensic bloodstain pattern analysis. Blood viscosity is important in these settings as it determines the driving pressure through biomedical devices and the shape of the bloodstain. However, animal blood can never exactly mimic human blood due to erythrocyte properties differing among species. This results in the species‐specific shear thinning behavior of blood suspensions, and it is therefore not enough to adjust the hematocrit of an animal blood sample to mimic the behavior of human blood over the entire range of shear rates that are present in the body. In order to optimize experiments that require animal blood, we need models to adapt the blood samples. We here offer mathematical models derived for each species using a multi linear regression approach to describe the influence of shear rate, hematocrit, and temperature on blood viscosity. Results show that pig blood cannot be recommended for experiments at low flow conditions (<200 s^−1^) even though erythrocyte properties are similar in pigs and humans. However, pig blood mimics human blood excellently at high flow condition. Horse blood is unsuitable as experimental model in this regard. For several studied conditions, sheep blood was the closest match to human blood viscosity among the tested species.

## Graphical abstract

Blood viscosity is crucial in the development of biomedical blood‐contacting devices, or forensic re‐enactment studies, and frequently, animal blood is used. Since the shear thinning of blood and the hematocrit‐temperature dependency of blood viscosity are species‐specific, our mathematical models derived from experimental data provide factors on how the hematocrit must be adjusted in a blood sample at hand to gain a desired viscosity value for human, pig, sheep and horse blood within a predefined shear rate range. These simple modifications allow running experimental setups at desired flow resistances.

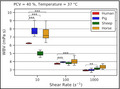

**New findings**

**Question**

Is pig blood indeed the best substitute for human blood in regard to blood viscosity, or are other large animals with contrasting RBC aggregability superior? What changes are necessary to match human blood viscosity?

**Finding**

Shear thinning of blood suspensions as well as the hematocrit and temperature dependency of blood viscosity are species‐specific. None of the investigated species accurately matched human blood viscosity over all the investigated ranges of shear rate, hematocrit, and temperature. With the help of mathematical models derived from the experimental data, we suggest changes of animal blood hematocrit and temperature to better match human blood viscosity.

**Importance**

Blood viscosity is crucial in the development of biomedical blood‐contacting devices, or forensic re‐enactment studies. Frequently, blood from large animals such as pigs is used in these tests. Knowledge about the limits of comparability to human blood is therefore essential.

## 1 INTRODUCTION

Animal blood often serves as a substitute for human blood, for example in the development of biomedical devices (Herbertson et al., 2015), in re‐enactment experiments of crime scenes (Sparer et al., 2020), or in the development of drug delivery systems (Namdee et al., 2015). Depending on the type of research blood samples are subject to a variety of different flow conditions. Ronco et al. used an experimental imaging technique to investigate the flow inside a hemodialysis membrane module and found average wall shear rates from 160 to 760 s^−1^ (Ronco et al., 2002). Higher deformation rates were found in an in vitro study of aortic valves (1700–2600 s^−1^) by laser Doppler velocimetry (Yap et al., 2012). Computational fluid dynamics are used to assess shear stresses in left ventricular assist devices. Depending on the type of the pump, wall shear stresses vary considerably, being low in pulsatile pumps (<500 s^−1^; Nanna et al., 2011) and high (up to 40,000 in rotor gap regions) in rotary pumps (Fraser et al., 2012). The test temperature might span from physiological conditions (37°C), to lower levels, for example, when simulating the effects of hypothermia treatment (Song & Lyden, 2012), or in forensic blood stain pattern analysis (Raymond et al., 1996).

Blood viscosity is the result of several features like red blood cell (RBC) aggregation and deformation, plasma viscosity, and RBC‐plasma coupling. Since it reflects the flow resistance, blood viscosity influences the outcome of experiments. In the testing of ventricular assist devices, it determines the pressure head (Herbertson et al., 2015), and in extra corporeal membrane oxygenator (ECMO) circuits (Pantalos et al., 2012) it influences the gas exchange across the hollow fiber membranes (Harasek et al., 2019). Even in applications where blood is subject to non‐physiological conditions, viscosity influences the results (Kim et al., 2016).

Ideally, the animal blood used in these applications should therefore be comparable to human blood viscosity, accurately mimicking magnitude, influence of shear rate, temperature, and hematocrit changes. Here, the dilemma comes into play that the extrinsic and intrinsic red blood cell (RBC) properties differ significantly among the species (Windberger, 2019) which modifies their coupling with the flow and thereby, whole blood viscosity (WBV) (Abkarian & Viallat, 2015). A comparison of important RBC properties for different species is given in Table 1. Increases in hematocrit—or packed cell volume (PCV)—increase viscosity due to the added solid fraction in the suspension. Aggregation (M_0_, M_1_) defines the tendency of RBCs to form rouleaux structures at low shear rates and therefore influences viscosity at these conditions. Differences are also apparent in the RBC size (mean corpuscular volume, MCV), and deformability (elongation index, EI_MAX_), which strongly influence the flow of RBCs through vessels (Abkarian & Viallat, 2015; Dipresa et al., 2021).

**TABLE 1:** Comparison of aggregation indices obtained by light transmission (M_0_ and M_1_), RBC elongation obtained by laser diffractometry (EI_MAX_) and packed cell volume (PCV) of four different species (Human, Pig, Sheep, Horse) (Windberger, 2019). Reference values for mean corpuscular volume (MCV), fibrinogen concentration (FIB), and PCV obtained from *(Kraft, 2005), **(Moritz, 2007), ***(Windberger et al., 2003 *)*

		Human	Pig	Sheep	Horse
MCV*	fL	76–100	50–68	23–48	37–55
FIB	mg dL^−1^	150–450***	160–390***	180–720**	150–330***
M_0_	‐	6 +/− 0.5	4 (2/5)	0	13 (12/15)
M_1_	‐	28 +/− 7	26 (21/31)	7 (3/9)	58 (44/68)
EI_MAX_	‐	0.62 (0.61/0.62)	0.63 (0.61/0.69)	0.47 (0.43/0.52)	0.68 (0.59/0.88)
PCV***	%	40–45	33–38	30.8 – 38	32–46

Based on these properties, animal blood must react differently to changes in shear rate, temperature or PCV than human blood. This observation was reported early by Stone et al. (1968) who investigated blood viscosity of human, sheep, goat, dog, and camel with PCV levels ranging from 25 to 70% and shear rates from 5 to 230 s^−1^. More recently a comprehensive comparison of nine mammalian species, including shear rate variations from 0.7 to 97 s^−1^, showed no uniform relation of WBV, plasma viscosity and erythrocyte aggregation, emphasizing the complexity of interspecies hemorheology (Windberger et al., 2003).

Considering the established differences, it is clear that no animal will exactly replicate human blood viscosity. This raises the question: Which animal blood is most suitable to be used as human blood substitute, that is, most accurately represents blood viscosity at a certain flow condition? Moreover, how can we adjust animal blood samples by simple and practicable means to better match human blood viscosity?

Therefore, we investigated whole blood viscosity of human, pig, sheep, and horse under a variety of different temperature, shear rate and PCV conditions. Pig was chosen as a species with comparable RBC aggregation and elongation to human blood and due to its use in forensic re‐enactment studies (Raymond et al., 1996) and *in vitro* testing (Lukitsch et al., 2021). Based on the properties given in Table 1, we expected that pig blood would show the best comparability with human blood. Horse and sheep were included due to their contrasting RBC aggregability and flexibility properties (Table 1). We formulated mathematical models from the experimental data that include the influence of shear rate, PCV, and temperature on whole blood viscosity from human, horse, sheep, and pig blood. Such models are available for human blood (Walburn & Schneck, 1976) (Eckmann et al., 2000); however, there is a distinct lack of mathematical viscosity models explicitly designed for animals. Used in their respective ranges, they allow an estimation of species‐specific whole blood viscosity, and aid in the adaptation of blood samples to match the human standard.

## 2 MATERIALS AND METHODS

### 2.1 Blood sampling

Blood samples were drawn from healthy human volunteers (*n* = 6, age: 23–33, BMI<30, non‐smokers, no intake of any medication for the last 7 days, 3 women, 3 men), horses (*n* = 10, age 8–20 years, 1 Warmblood, 4 Thoroughbreds, 5 Arabians, five mares, five stallions), pigs (*n* = 10, age: 4.5 months, Edelschwein, seven females, three castrated males), and sheep (*n* = 10, age: 1.5–3 years, female, Merino‐Wollschaf). Animal blood was drawn from the jugulary vein in horses and sheep and from the cranial caval vein in pigs. The procedure was approved by the institutional ethics and animal welfare committee and the national authority (BMWF‐66.009/0372‐WF/V/3b/2014 and BMWFW‐68.205/0188‐WF/V/3b/2015). Human blood was drawn from the antecubital vein after having obtained informed consent (approval of the ethical review committee of the Medical University of Vienna, EK No. 1892/2013). Blood was drawn using a 21‐gauge butterfly needle and a Vacuette blood collection system (Greiner Bio‐One GmbH, Kremsmünster, Austria), containing K_2_‐EDTA for anticoagulation. Blood was centrifuged (1500 g, 15 min) to separate plasma from RBC concentrate also including the buffy coat. Portions of blood plasma and concentrated RBC samples with varying hematocrit were mixed to generate new samples of defined packed cell volume (PCV; Hettich hematocrit centrifuge, Germany): 30%, 40%, 50%, and 60%. Tests were performed at the following temperatures (T): 37, 32, 27, 22, 17, and 12°C. All measurements were finished within a maximum of 10 h following blood withdrawal. Samples were stored at 7°C prior to their usage and gently mixed before the test system was filled with a new portion.

### 2.2 Viscosity measurements

A Physica MCR301 rheometer (Anton Paar, Austria) equipped with a Peltier controlled stainless steel double gap cylinder system (internal gap: 0.417 mm; external gap: 0.462 mm, cup length: 42 mm) was used. The Rheocompass^TM^ software (version 1.19.335, Anton Paar, Austria) was used for data acquisition. Isothermal strain‐controlled flow curves, at different shear rates between 10 s^−1^ and 1000 s^−1^ were created by starting at the highest shear rate to obtain shear viscosity. The sample remained in the measuring system until completion of the last flow curve. To remove any pre‐existing shear history, a 30‐second pre‐shear interval at 300 s^−1^ was set after each temperature equilibration. We started with the first flow curve at the highest temperature in human and horse to simulate cooling of blood during a clinical intervention, and at the lowest temperature in pig and sheep to simulate warming‐up blood that was stored in a fridge for forensic studies. A pilot study was performed to determine if the order of the temperatures at which the flow curves are measured affects the test outcome in human and horse blood samples. Native human (*n* = 6) and horse (*n* = 4) blood was used for a series of flow curve pairs starting at 7°C and finalizing the measurement at 37°C, as well as starting at 37°C and finalizing at 7°C, respectively. The 37°C flow curve was compared to identify differences in viscosity measurements obtained during increasing or decreasing the temperature. In human blood, both flow curves were congruent. This indicates that there is no difference in WBV, irrespective of the starting temperature point of the experimental setting. However, in horse blood, a sudden decrease of low shear WBV (at shear rates between 0.1 and 20 s^−1^) was observed at temperatures between 22 and 32°C, if the experiment started at the low temperature setting. WBV at shear rates below 21.5 indeed depended on the temperature, at which the measurements had been started.

### 2.3 Evaluation of experimental data

Preceding regression modeling, viscosity values were evaluated for each species as described in the following. Possible outliers were identified for each combination of shear rate, PCV and temperature by an IQR test. Therefore, the range between the lower (Q_1_) and upper (Q_3_) percentile of the data, or inter quartile range (IQR), was calculated. Observations below Q_1_‐1.5*IQR or above Q_3_+1.5*IQR were deemed as outliers and removed accordingly. Number of removed outliers and final sample sizes are given in Table 2.

**TABLE 2:** Model quality and ranges for shear rate, temperature, and PCV

		Human	Pig	Horse	Sheep
Final sample size	—	1289	2694	3762	2444
Nr. removed outliers	—	42	115	88	119
Range γ	s^‐1^	10–1000	10–1000	10–1000	10–1000
Range T	%	12–37	7–42	12−37	7 – 42
Range PCV	°C	20–60	30–60	30–70	30–60
RMSE mean	mPa.s	1.046	1.08	1.16	1.06
RMSE SD	mPa.s	0.004	0.003	0.004	0.004
$R_{\text{adj}}^{2}$	—	0.993	0.989	0.949	0.988

Differences between the species viscosity were evaluated at three different shear rates (10 s^−1^, 100 s^−1^, 1000 s^−1^) for all temperatures and PCVs. Primary, homogeneity of variance was asserted using Levene's test (Hastie et al., 2001). Based on Levene's test results, the Games–Howell post hoc test was applied to test for significant differences between the species, as it is applicable for unequal variances (Games & Howell, 1976). For all statistical tests, values of *p* < 0.05 were considered statistically significant. If no statistical difference was found between animal and human blood sample at a certain condition, it was deemed comparable at this state.

### 2.4 Modeling of viscosity

Mathematical models were created using a multiple linear regression (MLR) approach. Evaluation of sample sizes was done a‐priori using Greens Method (Green, 1991). Given *n* observations, the relationship between one dependent variable *y* (WBV) and multiple (p) independent variables x (shear rate, PCV, T) is given by Equation 1 (Hastie et al., 2001).

(1) $$\begin{array}{cc} & y_{i}=\beta_{0}+\beta_{1}x_{i,1}+\beta_{2}x_{i,2}+\beta_{3}x_{i,1}x_{i,2}+\cdots +\beta_{p}x_{i,p}+\epsilon \\ & \;\text{for}\;i=1,2,\;\ldots ,\;n \end{array}$$

The aim is to determine the regression coefficients β_i_ in a way that the error between the observation y_i_, and predicted value ${\hat{y}}_{i}$ is minimized (Equation 2). Values of β_i_ were obtained by an ordinary least squares (OLS) estimator.

(2) $$\sum_{i=1}^{n}\epsilon_{i}^{2}={\left(y‐X\beta \right)}^{T}\left(y‐X\beta \right)=\text{min}$$

Modeling procedure was equal for all species and consisted of three main steps:

Initial Fit: A logarithmic transformation of the dependent variable was performed prior to the fitting. Data were randomized and split into training (66% of data) and test (0.34% of data) sets. In order to account for non‐linear effects as well as interactions between the independent variables, which are a priori not known, the design matrix **X** was augmented by a variety of different functions (e.g., $\sqrt{\mathrm{PCV}},{\dot{\gamma }}^{2},\frac{1}{T},\ldots$). The resulting initial model was fit to the data, forming the basis for the next step. It must however be reduced to statistically significant variables, in order to avoid over‐fitting of the data.

Backwards Elimination: Model reduction was performed by a backwards elimination method. The entire data set was again randomized and split into training and test sets. Variables of the initial model were ranked by significance based on their respective p‐value. If the least significant variable exhibits a *p*‐ value above *p* > 0.0001, the variable was removed, and a new model created and fit to the training data. This process was repeated until the model contained only variables with statistically significant effects.

Cross Validation: Finally, the resulting reduced model was validated using a k‐fold cross validation method with 10 folds (Hastie et al., 2001). Therefore, the entire data set was randomized and split into 10 equally sized groups. One of the groups was designated as test data, while the nine remaining groups were used to train the model. Subsequently, the model error was calculated for both the training and test data set. As a measure of fit, the root mean square error (RMSE) is calculated (Eq. 3). This process was repeated for each unique group.

(3) $$\text{RMSE}=\sqrt{\frac{1}{N}\sum_{i=1}^{N}{\left(y_{i}‐\hat{y_{i}}\right)}^{2}}$$

The overall model quality was assessed by the adjusted coefficient of determination ($R_{\text{adj}}^{2}$) as it incorporates sample sizes and number of predictors (Hastie et al., 2001). It should be emphasized that the individual terms of the presented formulas do not constitute physical behavior but are means of describing the experimental data. Statistical evaluation, modeling, and figure creation were performed by Python 3.6 (Python Core Team, 2019) using the following packages: Modeling: pandas, numpy, statsmodels, pingouin, and sklearn, Figures: matplotlib and seaborn.

## 3 RESULTS

### 3.1 Experimental results

**FIGURE 1:**
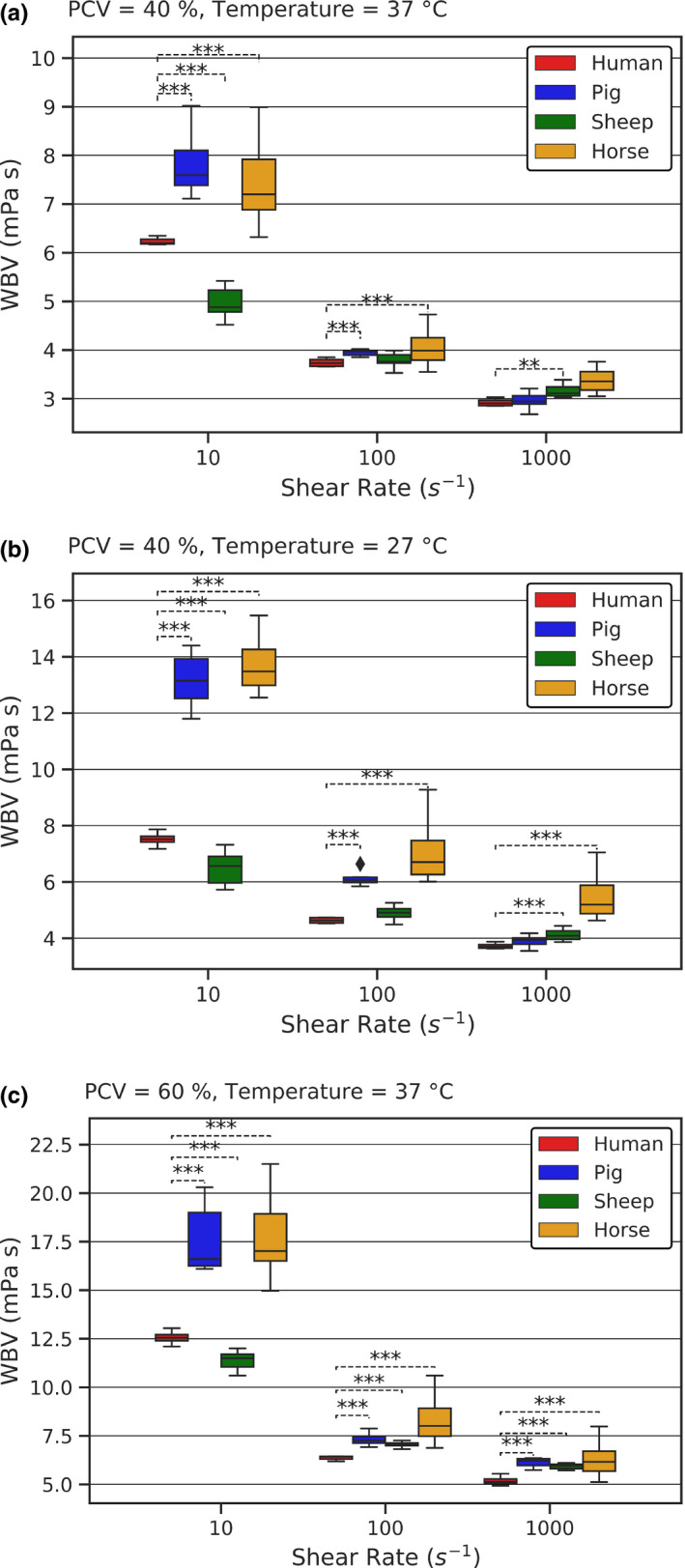
Experimental results for selected combinations of shear rate, PCV, and temperature. (a) Experimental results for physiological conditions. (b) Experimental results for decreased temperature. (c) Experimental results for increased PCV. Boxes shifted horizontally to avoid overlapping. Brackets indicate significant differences (**p < 0.05, ***p < 0.01) between human and other species

Generally, the species dependency of WBV diminishes with increasing the shear rate but becomes augmented by raising the PCV and by lowering the temperature. Figure 1 summarizes WBV for selected combinations of PCV and temperature (T) at three different shear rates (10 s^−1^, 100 s^−1^, 1000 s^−1^), with Figure 1a corresponding to physiological conditions (40% PCV, 37°C), Figure 1b to decreased temperature (40% PCV, 27°C), and Figure 1c to increased PCV levels (60% PCV, 37°C). For clarity, significant differences between the groups are only depicted between human and animal species in the graphs. Data of Figure 1 are additionally given in a tabular format, including mean values and standard deviations (Table S1).

Shear thinning was observed in all blood samples, with all species showing near‐Newtonian behavior at shear rates above 200 s^−1^. Significant differences compared to human were present in every blood type at low shear rate (10 s^−1^), while pig and horse WBV were comparable to each other (Figure 1a). Judging by the mean values, sheep showed the least, and equine the biggest, deviation from human WBV. Overall, the highest WBV is found in horse and pig blood, followed by human and ultimately sheep blood (Table S1). This ranking holds true for decreased temperature (Figure 1b) and for increased PCV (Figure 1c), as well.

Increasing the shear rate to 100 s^−1^ reduced the differences between the species but did not abolish the differences compared to human. An exemption is sheep WBV that became comparable with human WBV at normal condition and at decreased temperature. Equine WBV was the highest, showing the biggest deviation from human WBV, followed by pig, and sheep WBV. At the highest shear rate (1000 s^−1^), pig and human WBV became now comparable at normal condition and at decreased temperature, while sheep WBV became higher than human WBV. Horse WBV remained elevated throughout. Generally, horse WBV displayed the highest values in almost all conditions and was least comparable with human blood, whereas sheep and pig blood showed distinct comparability with human blood at certain conditions.

**FIGURE 2:**
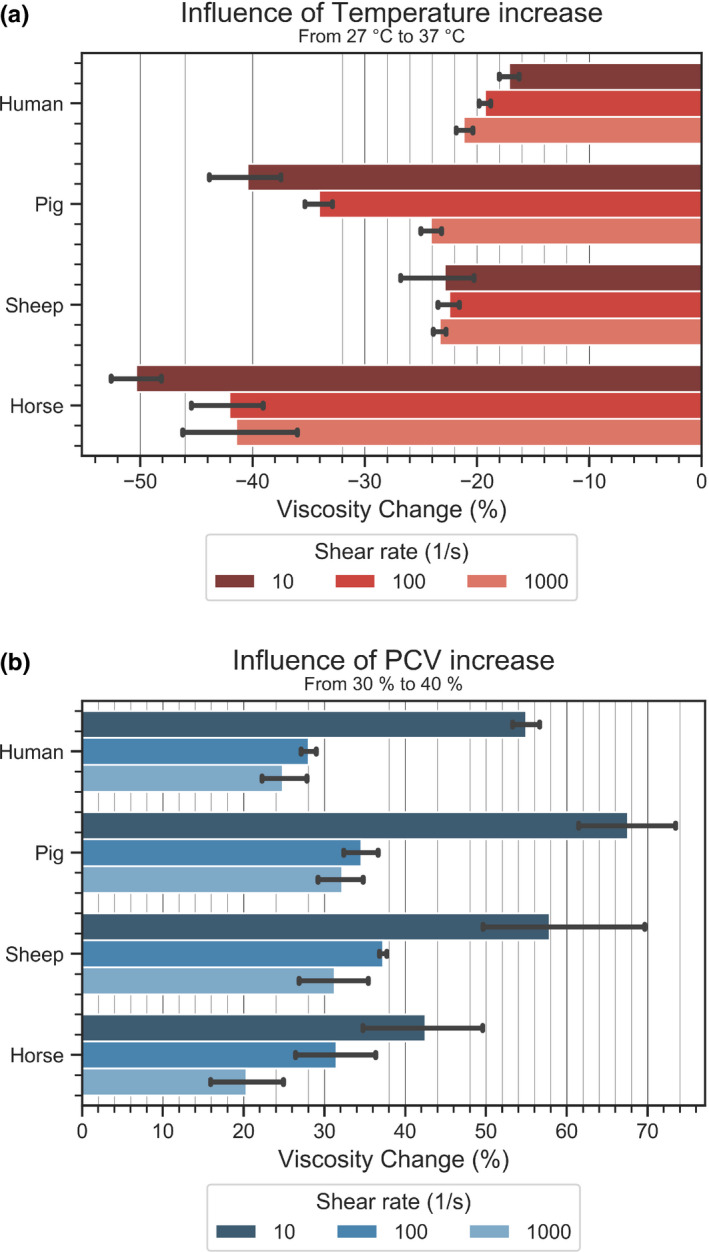
(a) Change of WBV at a temperature increase from 27°C to 37°C for three different shear rates at PCV = 40%; (b) Change of WBV at a PCV increase from 30% to 40% at three different shear rates at T = 37°C. Error bars denote standard deviation

In order to highlight the influence of temperature and PCV on whole blood viscosity, we calculated the percentage viscosity change for an increase in temperature or PCV at three different shear rates. The effects of a temperature increase from 27°C to 37°C are given in Figure 2a. Human and sheep blood samples showed a similar WBV decrease (about 20%) when the temperature rose. The highest temperature dependency is found in horse, followed by pig, human, and sheep blood. Similarly, the WBV changes for a PCV increase from 30% to 40% at constant temperature are given in Figure 2b. Elevating the PCV also enlarged the differences between the species. Notably for all species, lower shear rates lead to a larger increase in WBV. At 10 s^−1^ the highest PCV dependency is found in pig, followed by sheep, human and horse.

### 3.2 Modeling results

Inspection of the residuals revealed no clear trend for any of the models. Notably, we found that, across all species the biggest deviations between the experimental and calculated data occurred at high viscosities, that is, low temperature, low shear rate, and high PCV values. Model quality and valid range of the independent variables for each of the respective species are summarized in Table 2. Mean and standard deviation (SD) of the RMSE acquired by the cross validation are presented. Based on the adjusted coefficient of variation, the model for human blood demonstrates the best overall fit, followed by pig, sheep, and horse. If applied in their respective ranges, the models presented in this work are capable of accurately predicting WBV over a wide range of different conditions, including the effect of shear rate, temperature, and PCV changes.

Following are the model equations for the estimation of the WBV (*η*). Values of the coefficients (*C_α_*) are given in the supporting information (Table S2).

Human Model

(4) $$\begin{array}{cc} ln\left(\eta \right) & =C_{1}\text{PCV}+C_{2}\gamma +C_{3}T‐C_{4}\sqrt{\gamma }‐C_{5}\sqrt{T}+C_{6}e^{1/\text{PCV}}\\ & \;+C_{7}e^{1/\gamma }‐C_{8}e^{1/T}‐C_{9}e^{1/\text{PCV}\gamma }+C_{10} \end{array}$$

Porcine Model

(5) $$\begin{array}{cc} ln\left(\eta \right) & =\left(C_{1}ln\left(\mathrm{PCV}\right)ln\left(\gamma \right)‐C_{2}\right)ln\left(T\right)+C_{3}\text{PCV}‐C_{4}\gamma ‐C_{5}T‐C_{6}\sqrt{\text{PCV}}\\ & \;+C_{7}\sqrt{\gamma }‐C_{8}e^{1/\left(\text{PCV}+\gamma \right)}+C_{9}e^{1/\left(\gamma +T\right)}+C_{10}ln\left(\text{PCV}\right)‐C_{11}ln\left(\gamma \right)+C_{12} \end{array}$$

Ovine Model

(6) $$\begin{array}{cc} ln\left(\eta \right) & =C_{1}\text{PCV}+C_{2}\gamma +C_{3}T‐C_{4}\sqrt{\gamma }‐C_{5}\sqrt{T}+C_{6}e^{1/\text{PCV}}\\ & \;+C_{7}e^{1/\gamma }‐C_{8}e^{1/\text{PCV}\gamma }‐C_{9}e^{1/\text{PCV}T}+C_{10}e^{1/\text{PCV}T\gamma }‐C_{11} \end{array}$$

Equine Model

(7) $$\begin{array}{cc} ln\left(\eta \right) & =\left(\left(‐C_{1}ln\left(\mathrm{PCV}\right)+C_{2}\right)ln\left(\gamma \right)+C_{3}ln\left(\text{PCV}\right)‐C_{4}\right)ln\left(T\right)+C_{5}\text{PCV}‐C_{6}\gamma ‐C_{7}T\\ & \;‐C_{8}\sqrt{\text{PCV}}+C_{9}\sqrt{\gamma }+C_{10}ln\left(\text{PCV}\right)‐C_{11}ln\left(\gamma \right)+C_{12} \end{array}$$

**FIGURE 3:**
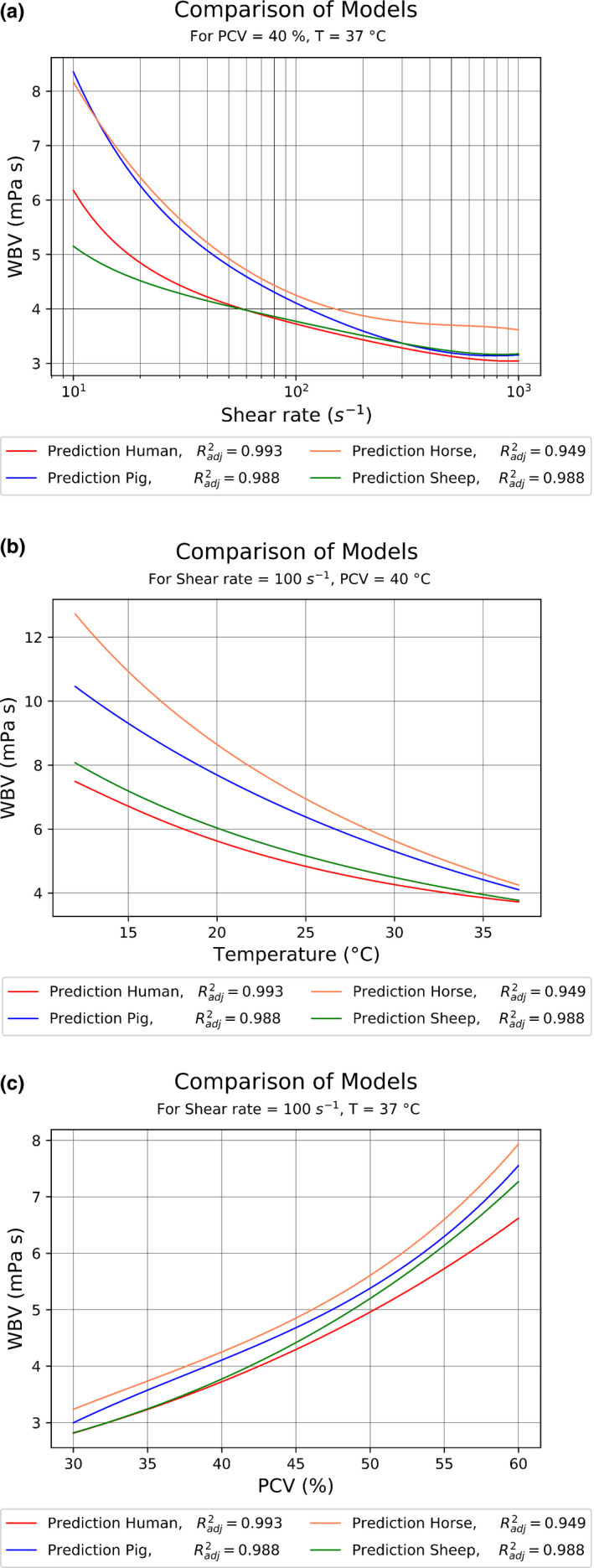
Modeling results for selected combinations of shear rate, temperature, and PCV. Graphs show the influence of shear rate (a), temperature (b), and PCV (c). The adjusted coefficient of determination is given as a measure of fit to the experimental data

Based on the MLR models (Eq. 4−7), the influence of the three independent variables on WBV was evaluated and plotted in Figure 3.

Finally, the models at hand allow an estimation of the changes needed to match animal to human WBV by either adjusting temperature or PCV. Exemplary, Table 3 presents the calculated adjustments to porcine or ovine blood in order to match human blood viscosity at PCV = 40% and T = 37°C.

**TABLE 3:** Calculated temperature or PCV changes needed in order to match human WBV at PCV = 40% and T = 37°C at three different shear rates (*blood instability due to temperature >45°C)

	Adjustment by PCV [%]		Adjustment by Temperature [°C]	
	Pig	Sheep	Pig	Sheep
10 s^−1^	33.8	44.5	n.a.*	29.6
100 s^−1^	35.5	39.5	40.9	37.9
1000 s^−1^	40.7	38.7	36.5	38.7

## 4 DISCUSSION

### 4.1 Shear rate dependency

One aim of this study was to show the application limits of animal blood as a human blood substitute. We started with pig blood because due to comparable RBC features it is often used to substitute human blood in mock circulations or re‐enactment studies. We expected comparability at all shear rates in PCV‐matched samples because RBC properties are similar between pigs and humans (compare with Table 1). However, at the lower shear rates pig blood viscosity was higher than human blood viscosity and reached even values like obtained for horse blood. The high viscosity of horse blood can be explained by the ability of RBCs to generate strong rouleaux, but this is not the case in pig blood (Windberger et al., 2003). Rouleaux formation is therefore not the only cause for high blood viscosity at low shear rates. This assumption is supported by observations on rat blood. There is almost no RBC aggregation in rat blood (Baskurt et al., 1997), but blood viscosity and shear thinning are profound (Windberger, 2019). To find an explanation for the behavior at low shear rates, we exposed the same samples to small amplitudes of oscillating shear stress, to test them at minimal disturbance by the imposed shear field (Sparer, 2016). Such conditions occur in the axial streamline where shear rates approach zero, considering a blunted parabolic flow profile in small vessels and veins. We found that pig blood exerted higher storage moduli (G′) than any other species tested so far (Supporting Information, Figure S1). We also found that at 60% HCT (and at lower temperature even at 50% HCT), the EDTA‐anticoagulated pig blood samples behaved gel‐like. This shear elasticity was of course weak, but the lower the temperature became, the more projected G′ above G″, and the stronger became the gel structure (Supporting Information, Figure S2). This might be relevant for re‐enactment studies that must be performed at ambient temperature. Pig blood is therefore of limited use if comparability with human blood is needed at low flow velocities or shear rates. This poses also a potential limitation for the testing of low‐flow membrane oxygenators, where recent numerical studies suggest average wall shear rates well below 200 s‐1, and potential stagnating zones (Dipresa et al., 2021). At these conditions, even sheep blood would be superior to pig blood because blood viscosity matched best between sheep and human.

At high shear rates, porcine blood is a suitable substitute for human blood at matched PCV value. Pig blood became increasingly comparable to human blood as shear rates increased, while sheep blood became increasingly different. In smaller vessels or in biomedical devices where high shear rates are expected (Fraser et al., 2012), pig blood becomes therefore superior to sheep blood. Such flow conditions are typically found in ventricular assist devices (Fraser et al., 2012) and even in membrane oxygenators for adult patients (Zhang et al., 2007). The higher sheep blood viscosity at 1000 s^−1^ can be attributed to the low surface to volume ratio of RBCs, leading to a more rounded shape, which makes cell deformations more difficult. This relative stiffness has consequences for the distribution of RBCs in the vessel cross‐section (Kumar et al., 2014) and might not allow that kind of “blood structuring” known for human blood (Mchedlishvili et al., 1997), and results in higher flow resistance and WBV.

The comparison between sheep and horse shows how much RBC aggregability compromises the comparability to human blood. Higher RBC aggregation than the human values shifted WBV farther away from the human value than lower RBC aggregation and classifies horse blood as completely unsuitable as a substitute for human blood.

### 4.2 Temperature and PCV dependency

While 37°C is the temperature of choice in physiological studies, in certain applications blood will be tested at lower or higher levels. To warm up blood might not be always feasible, and eventually the performance of biomedical devices is examined under hypothermic conditions (Kirkpatrick et al., 2003). Blood stains during a crime can form at other temperatures than found in a body (Larkin & Banks, 2013). We therefore included testing the temperature profile of WBV and found that horse WBV showed the highest temperature dependency, followed by pig and human/sheep. Since human and sheep WBV showed a similar temperature dependency, cooling or warming samples during an experiment would change their WBV in the same way.

The PCV dependency of WBV was reported only for human blood samples (Brooks et al., 1970; Stone et al., 1968) and is now supplemented by animal data. PCV variations from 30% to 60% revealed a species‐specific increase in blood viscosity with pig showing the highest dependency followed by sheep, human, and ultimately, horse (Figure 2b). Contrary to temperature, the impact of PCV changes on WBV was higher at low shear rates than at high shear rates. The closest match to human was again found with sheep.

Matching of blood viscosity at a macroscopic scale cannot be equaled to matching blood rheology in small conduits. The macroscopic view on blood as a liquid might be sufficient to calculate the pressure drop or the flow distribution in a certain biomedical device. But at smaller vessel diameters the effect of the wall on the flowing suspension becomes pronounced (Thurston, 1976), so that the different blood cells arrange in the vessel cross‐section according to their size and stiffness (Kumar et al., 2014). At the latest then blood is no more a homogenous material. Further difficulties exist when the flow is unsteady, like in arteries (Reneman et al., 2006), even though the vessel diameter is large. Even turbulent flow at low Reynolds numbers was postulated to occur in arteries as a result of the oscillations occurring there (Saqr et al., 2020). Averaged shear rates close to the wall of the carotid artery were found in the range of 200–500 s^−1^, but systolic shear rates can be 1000 s^−1^ and even higher (Panteleev et al., 2021). This quick change of shear rates at one spot makes blood rheology a very dynamic process.

When RBC properties change, it adds a further level of complexity to flow simulations. This is the case when animal blood is used as a substitute for human blood. Species‐specific features, including but not restricted to those given in Table 1, will influence the flow behavior of blood. One could say now that one should only work with human blood. But the use of human blood is not always an option. Matching blood viscosity is still preferable to using animal blood as withdrawn.

We have shown that matching the PCV value between human and animal blood is not enough to derive the identical WBV value over the entire range of shear rates present in a body, because the shear thinning behavior is species‐specific. We went on to our second objective to identify what PCV level in a pig, sheep and horse blood sample needs to be adjusted in order to derive the desired WBV level within the shear rate ranges in an application. Our models also allow including many test temperatures (12 – 37°C) into this match. Generally, any desired human WBV can be achieved. The work to be done is to first identify the representative shear rate and test temperature, and afterward adjust the animal blood sample present to the calculated PCV value.

This work did not include all mammalian species that are potential substitutes for human blood. Nevertheless, one must expect that no species will precisely match human blood viscosity over a wide range of conditions, and adjustments will always be necessary.

## COMPETING INTERESTS

None declared.

## AUTHOR CONTRIBUTIONS

Conception or design of the work: P.E. and U.W. Acquisition, analysis, or interpretation of data for the work: All authors. Drafting of the work or revising it critically for important intellectual content: All authors. All authors approved the final version of the manuscript and agree to be accountable for all aspects of the work in ensuring that questions related to the accuracy or integrity of any part of the work are appropriately investigated and resolved. All persons designated as authors qualify for authorship, and all those who qualify for authorship are listed.

## Supporting information



Supplementary Material

## Data Availability

The data that support the findings of this study are available upon request.
