# Supplementary material for: Animal blood in translational research: How to adjust animal blood viscosity to the human standard
Source: Physiol Rep. 2021 May 27;9(10):e14880. doi: 10.14814/phy2.14880 (PMC8157792; doi:10.14814/phy2.14880)

**Supporting Information**

Table S1:

Experimental results represented as mean whole blood viscosities at shear rates of 10, 100, and 1000 s^-1^, and selected temperatures (T) and packed cell volumes (PCV). Standard deviation is given in brackets. Bold values denote the species that most closely resembles human blood viscosity at this condition.

| $\mathrm{WBV}\left[ \mathrm{mPas} \right]$ | **Human** | **Pig** | **Sheep** | **Horse** |
| --- | --- | --- | --- | --- |
| For T = 37°C and PCV = 40 % | | | | |
| 10 $s^{-1}$ | 6.2 (0.08) | 7.8 (0.64) | **5.0 (0.3)** | 7.4 (0.75) |
| 100 $s^{-1}$ | 3.7 (0.08) | 3.9 (0.10) | **3.8 (0.15)** | 4.0 (0.31) |
| 1000 $s^{-1}$ | 3.05 (0.07) | **3.0 (0.17)** | 3.2 (0.14) | 3.4 (0.27) |
| For T = 27°C and PCV = 40 % | | | | |
| 10 $s^{-1}$ | 7.5 (0.24) | 13.2 (0.88) | **6.5 (0.56)** | 13.7 (0.92) |
| 100 $s^{-1}$ | 4.6 (0.10) | 6.1 (0.24) | **4.9 (0.24)** | 7.1 (1.11) |
| 1000 $s^{-1}$ | 3.7 (0.10) | **3.9 (0.19)** | 4.1 (0.21) | 5.5 (0.82) |
| For T = 37°C and PCV = 60 % | | | | |
| 10 $s^{-1}$ | 12.6 (0.33) | 17.5 (1.58) | **11.4 (0.46)** | 17.8 (1.78) |
| 100 $s^{-1}$ | 6.4 (0.12) | 7.3 (0.29) | **7.1 (0.14)** | 8.3 (0.99) |
| 1000 $s^{-1}$ | 5.2 (0.23) | 6.1 (0.23) | **5.9 (0.14)** | 6.3 (0.73) |

Table S2:

Model Constants for Equations 4 -7

Human Model (Eq. 4)

| C_1_ | C_2_ | C_3_ | C_4_ | C_5_ | C_6_ | C_7_ | C_8_ | C_9_ | C_10_ |
| --- | --- | --- | --- | --- | --- | --- | --- | --- | --- |
| 0.02977 | 0.0004283 | 0.06059 | 0.0254 | 0.9934 | 3.393 | 7.547 | 6.529 | 155.525 | 155.304 |

Porcine Model (Eq. 5)

| C_1_ | C_2_ | C_3_ | C_4_ | C_5_ | C_6_ | C_7_ | C_8_ | C_9_ | C_10_ | C_11_ | C_12_ |
| --- | --- | --- | --- | --- | --- | --- | --- | --- | --- | --- | --- |
| 0.01622 | 0.329 | 0.511 | 0.0003658 | 0.03464 | 12.599 | 0.07254 | 54.386 | 5.2489 | 20.052 | 1 | 41.556 |

Ovine Model (Eq. 6)

| C_1_ | C_2_ | C_3_ | C_4_ | C_5_ | C_6_ | C_7_ | C_8_ | C_9_ | C_10_ | C_11_ |
| --- | --- | --- | --- | --- | --- | --- | --- | --- | --- | --- |
| 0.0364 | 0.0004653 | 0.009961 | 0.0267 | 0.419 | 11.88 | 5.749 | 172.697 | 64.032 | 224.039 | 2.94258 |

Equine Model (Eq. 7)

| C_1_ | C_2_ | C_3_ | C_4_ | C_5_ | C_6_ | C_7_ | C_8_ | C_9_ | C_10_ | C_11_ | C_12_ |
| --- | --- | --- | --- | --- | --- | --- | --- | --- | --- | --- | --- |
| 0.0260 | 0.174 | 0.4482 | 2.2519 | 0.3746 | 0.0008468 | 0.0331 | 8.685 | 0.0796 | 12.321 | 0.7684 | 0.8552 |

**Figure S1:** Storage (G´) and loss (G´´) moduli of pig, horse, and human blood (40% PCV, 37°C) at increasing amplitudes of small shear stress (SAOS) in double gap cylinder geometry (internal gap: 0.417mm; external gap: 0.462mm, cup length: 42mm). Pig blood shows the highest shear elasticity (G´) among these species. Horse RBCs show the highest RBC aggregability, but not the highest G´-values.


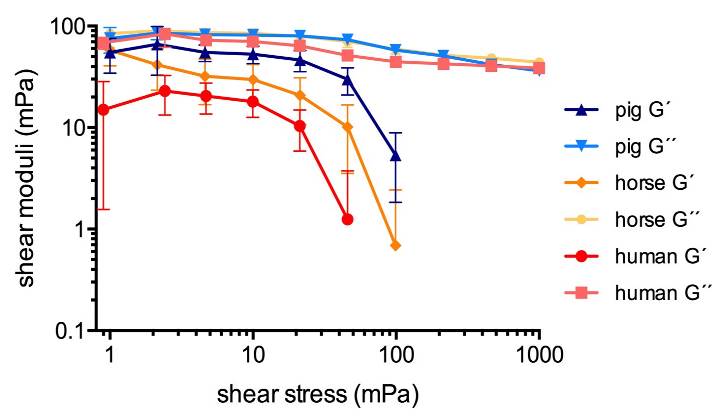


**Figure S2:** Storage (G´) and loss (G´´) moduli of pig blood suspensions with 40, 50, and 60% PCV in linear mode in the double gap cylinder system. Data obtained at 22 and 37°C. At physiologic body temperature and at 60% PCV G´ = G´´, indicating pig blood as a gel. Solid-like behavior is pronounced at lower temperatures.


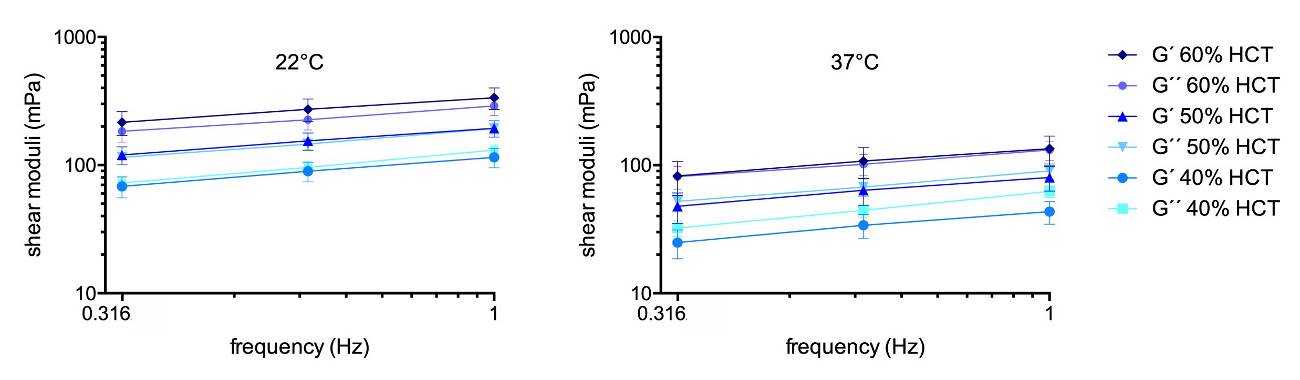

Supplement: Supplementary file 1 — Supplementary Material [file PHY2-9-e14880-s001.docx]
